# Training with ultrasound-guided puncture simulator of arteriovenous fistula for hemodialysis: experience from a center of excellence in Pará, Brazil

**DOI:** 10.1590/1677-5449.202401842

**Published:** 2026-03-16

**Authors:** Elisa Maria Novaes Barros, Glauco dos Santos Melo, Murilo Vasconcelos de Oliveira, Victor Hugo Guerreiro Americo Gomes, Gabriel Henrique de Paiva Ramos, Silvio Jorge de Oliveira Bentes, Humberto Balbi Reale, José Maciel Caldas dos Reis

**Affiliations:** 1 Fundação Hospital de Clínicas Gaspar Vianna – FHCGV, Belém, PA, Brasil.; 2 Hospital Beneficente Portuguesa – HBP, Belém, PA, Brasil.

**Keywords:** ultrasonography, simulation exercise, arteriovenous fistula, dialysis, nursing

## Abstract

**Background:**

Arteriovenous fistulas (AVF) and arteriovenous grafts are important arteriovenous accesses (AVA) for patients who need hemodialysis. However, in approximately 20 to 40% of AVFs, professionals fail to successfully cannulate a new AVA for consistent use. Therefore, the ability to perform ultrasound-guided puncture is a necessary skill, particularly for accesses classified as 'difficult'. However, only a minority of nursing professionals consider themselves capable of handling ultrasound. Therefore, simulated practice environments can enable trainees to develop the skills and dexterity needed to simultaneously handle the ultrasound probe and insert the needle.

**Objectives:**

The object of this study was to analyze the effects of training with an ultrasound-guided arteriovenous fistula access simulator at a center of excellence in Belém, PA, Brazil.

**Methods:**

This analytical intervention study assessed a nursing team (17 nurses) before and after training in ultrasound-guided puncture using a simulator model comprising a chicken breast and a latex balloon, chosen because of its low cost and high fidelity. Training was carried out on January 26th and 27th, 2024, at the Fundação Hospital de Clínicas Gaspar Vianna.

**Results:**

After training with a simulator, participants’ performance improved in all analyzed variables, with statistical significance differences. Furthermore, they rated the model as a good simulation of reality and as important for professional training and reported feeling more confident after training.

**Conclusions:**

Training with the ultrasound-guided puncture simulator positively influenced the performance of hemodialysis professionals.

## INTRODUCTION

Arteriovenous fistulas (AVF) are essential arteriovenous accesses (AVA) for dialysis patients and an autologous AVF is considered the ideal vascular access for long-term use.^1,2^

Failure to cannulate a new AVA and thereby enable its consistent use, which ideally should be catheter free, occurs in approximately 20 to 40% of AVFs. This is primarily caused by thrombosis and failure to mature, while failure of the first cannulation attempt, ischemia, and infection are among the less frequent causes.^3-7^

The patient’s experience during AVA cannulation is a crucial factor in sustained and effective use of the access thereafter. As such, patients who need multiple needle insertion attempts or who suffer severe infiltration, bleeding, or hematoma formation have high levels of dissatisfaction and prolonged dependence on central venous catheters, coupled with higher medical care costs because of additional diagnostic interventions and tests.^8^

Ultrasonography (USG) is an imaging method that offers several advantages: it is noninvasive, it is relatively low cost, and it does not need iodinated contrast or radiation in routine use. However, efficacy is limited because it is an operator-dependent technique. In this scenario, USG-guided cannulation, especially for placement of catheters, is already considered to be supported by IA level evidence.^9^ However, in the context of AVF puncture, it is more likely to be employed in cases considered difficult, which can include a new AVF in an elderly patient, an access with a history of multiple cannulation attempts, a small caliber vessel, proximity to structures such as arteries or nerves, or a vessel for which cannulation at the first attempt is critical.^10^ However, within nephrology, particularly as practiced in Brazil, integration of USG into clinical assessment is still rarely adopted. The main barriers to adoption are the cost of the equipment and lack of time available to learn how to use USG.^11^

Effective use of USG is dependent on the operator receiving the appropriate training, which includes basic knowledge and theoretical and practical instruction from experienced operators on how to handle the equipment and on cannulation procedures. Formal training, as part of the renal nursing curriculum, must culminate in successful use of USG and ensure patient safety.^12^ In this context, a simulated practical environment enables trainees to develop their skills and acquire the dexterity needed to simultaneously handle the USG probe and insert the needle. It has already been demonstrated that training with simulators improves the results of health care education and is valuable for other aspects of vascular cannulation.

Additionally, it is also important to point out that care for chronic kidney disease patients faces specific problems in the Amazon region, such as low educational level, inadequate income, preexisting diseases, and difficulties affecting access to health services, which constitute critical vulnerabilities.^13^ Adoption of modern technologies, such as USG for AVF puncture and other procedures in hemodialysis departments shows that, despite these challenges affecting logistics and infrastructure, excellent care services can still be provided in the region.

In this study we analyzed the impact of providing training with a model simulating ultrasound-guided AVF access at a center of excellence in Belém, Pará, Brazil.

## METHOD

### Study design

This was a cross-sectional, observational, descriptive, and analytical study. The methodology employed adheres to the precepts set out in the Good Publication Practice Guidelines, developed by the Committee on Publication Ethics.

**Figure 1:**
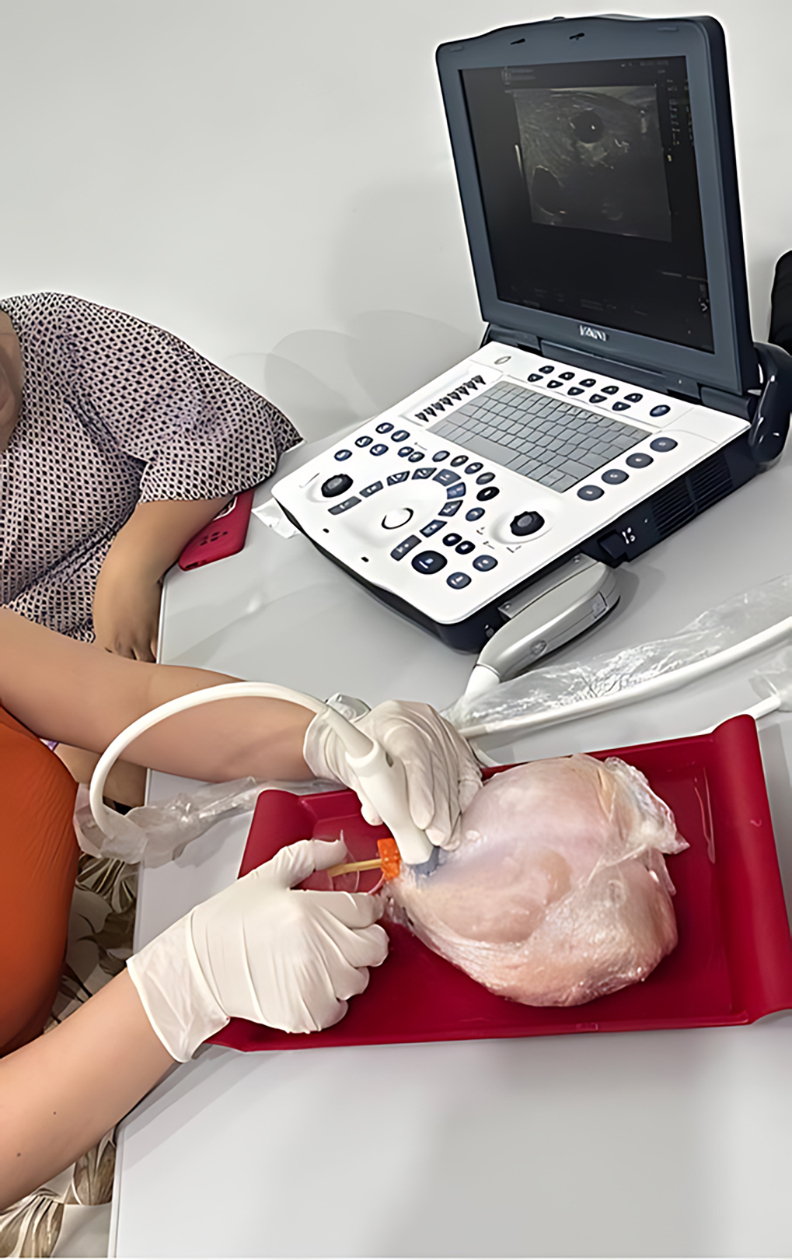
Training nursing professionals from the Department of Renal Replacement Therapy, Fundação Hospital de Clínicas Gaspar Vianna, in ultrasound-guided puncture. Source: authors’ files.

During the intervention, data were collected on members of a nursing team (17 nurses) before and after they underwent a practical training course in ultrasound-guided puncture using models simulating hemodialysis AVFs, on the 26th and 27th of January, 2024, in the Department of Renal Replacement Therapy (DRRT) at the Fundação Hospital de Clínicas Gaspar Vianna (FHCGV), in the city of Belém, Pará, Brazil (Figure 1)**.**

### Ethical considerations

This project was approved by the FHCGV Research Ethics Committee and registered on the Plataforma Brazil under Ethics Appraisal Submission Certificate 48063521.0.0000.0016 and opinion number 5.412.470. All study participants signed a free and informed consent form before taking part in the study, as shown in Appendix A.

### Study population

**Figure 2:**
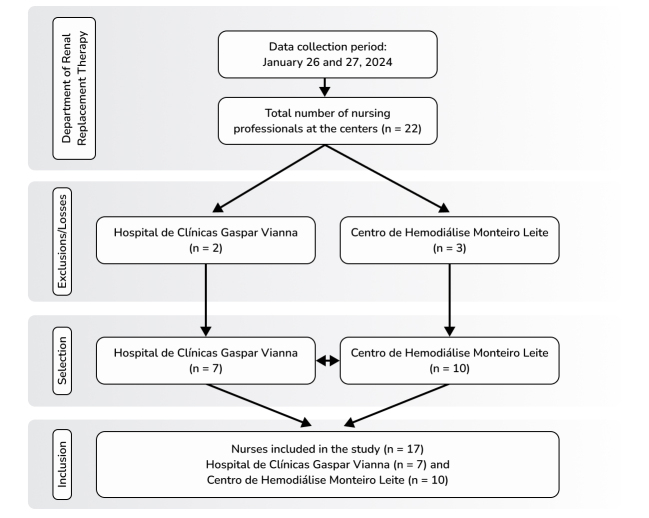
Flowchart illustrating inclusion of professionals in the study. Source: the authors.

All professionals from the FHCGV DRRT who voluntarily expressed an interest in taking part in puncture simulation training were enrolled. Professionals were excluded if they did not give consent or if they did not work with dialysis patients, as illustrated in the Strengthening the Reporting of Observational Studies in Epidemiology flowchart, shown in Figure 2.

**Figure 3:**
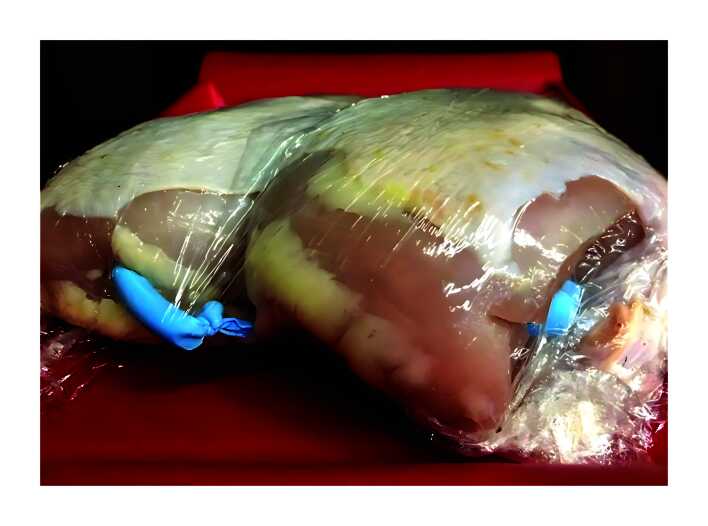
Puncture model comprising chicken breast and tubular latex balloon filled with ultrasound gel. Source: authors’ files.

**Figure 4:**
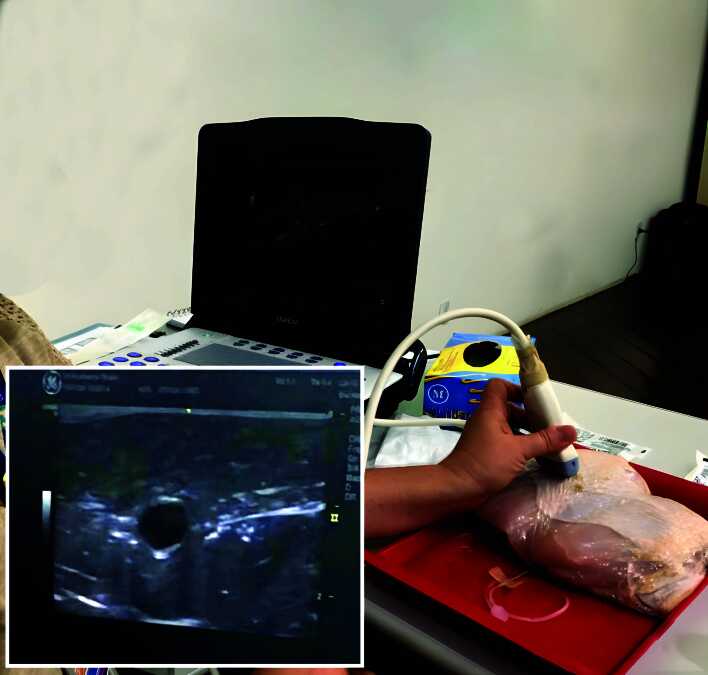
Ultrasonographic image of the puncture model. Source: authors’ files.

The training model is a realistic, non-human, experimental model comprising a chicken breast with skin and a latex balloon containing USG gel, wrapped in plastic film (Figure 3). The USG machine was used to acquire images of a tubular structure, with anechoic and homogenous content within the musculature of the chicken breast, which has a similar ultrasound density to human muscle tissue and diameters compatible with those found in AVFs among the dialysis patient population (Figure 4), thus reliably simulating the ultrasonographic images obtained in humans.

### Sample size calculation

In order to describe the impact of training with a realistic simulation of ultrasound-guided AVF access at a center of excellence in Belém, a sample size calculation for the population was performed considering a 95% confidence level (Z = 1.96), acceptable error of 5%, and expected proportion of 50%. Based on the size of the nursing team (DRRT nurses), comprising a total of 22 professionals in the department, the ideal sample size would be approximately 21 participants. However, it is important to contextualize the study design: this is an intervention study involving training of a specific, closed group of professionals, that is not targeting generalization of the results to other populations, but seeking to analyze the direct effects of the intervention within the local setting of the department being studied. From this perspective, the sample of 17 participants equates to 77% of the total population of interest, which confers a quasi-census character. The calculation employed methodology proposed by Luchesa & Chaves in 2011.^14^

### Intervention

Each study participant acts as their own control, i.e., each participant’s performance is compared before and after training. The data on initial performance on the simulator were compared with data from a final assessment, in line with the study’s dynamics, which was split into two phases, and involved an initial theoretical component, an immersive practical course, a final skills assessment and a later knowledge retention assessment.

The first phase was a theoretical and practical course lasting 2 days. Trainees were initially given the opportunity to perform ultrasound-guided punctures autonomously (initial data), then they were monitored by experienced specialists and given feedback, making changes to the process steps of the procedure, and then, finally, they performed the procedure autonomously once more (final data). At the end of the course, research protocols were filled out with the variables to be analyzed (Appendices B, C and D), which included:

1. Demographic data;
2. Assessment of DRRT professionals’ previous experience with ultrasound-guided puncture of AVFs;
3. Professionals’ opinions on training with the simulator;
4. Professionals’ performance before and after training with the simulator.

Later, 10 months after the course had been run, another questionnaire was administered to assess long-term knowledge retention among the professionals and make a subjective analysis of the impact of the course on the participants’ professional routines (Appendix E).

### Statistical analysis

Data were tabulated in Excel spreadsheets and the characteristics of the study population were described using percentages for demographic variables. The Wilcoxon statistical test was applied, with p values < 0.05 considered statistically significant.

## RESULTS

**Figure 5:**
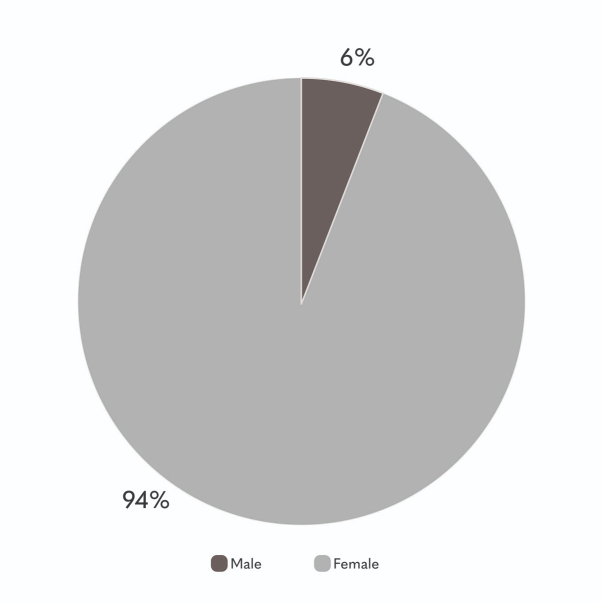
Sex distribution of professionals from the Department of Renal Replacement Therapy, Fundação Hospital de Clínicas Gaspar Vianna, trained in arteriovenous fistula puncture. Belém, Pará, Brazil, 2024 Source: the author.

A majority of the study participants were female, as illustrated in Figure 5. As shown in Table 1, 65% of the professionals were in the age bracket from 33 to 45 years or older, with a mean age of 28.8 years.

**Table 1:** Age distribution of professionals from the Department of Renal Replacement Therapy, Fundação Hospital de Clínicas Gaspar Vianna, trained to perform arteriovenous fistula puncture. Belém, Pará, Brazil, 2024.

**Age bracket**	**Number**	**Percentage**
27 to 33	6	35%
33 to 39	1	6%
39 to 45	3	18%
45 or older	7	41%
Total	17	100%

Source: the author.

The nurses’ mean time in the profession was 12.3 years, indicating that on average these professionals had more than a decade of experience in the job. However, the standard deviation was 8.4 years, indicating a relatively wide dispersion of the data. In other words, there was a large variation in the professionals’ experience, with some having very little experience and others having been working for many years. The least experience was 6 months, while the most was 25 years, revealing a wide range of experience among the professionals (Table 2).

**Table 2:** Measures of central tendency and dispersion of data on professionals from the Department of Renal Replacement Therapy, Fundação Hospital de Clínicas Gaspar Vianna, trained in arteriovenous fistula puncture, by time in the job in years. Belém, Pará, Brazil, 2024.

**Variable**	**Mean**	**Median**	**Standard deviation**	**Minimum**	**Maximum**
Time working with AVF punctures (years)	12.3	14.0	8.4	0.6	25.0

AVFs = arteriovenous fistulas. Source: the author.

**Figure 6:**
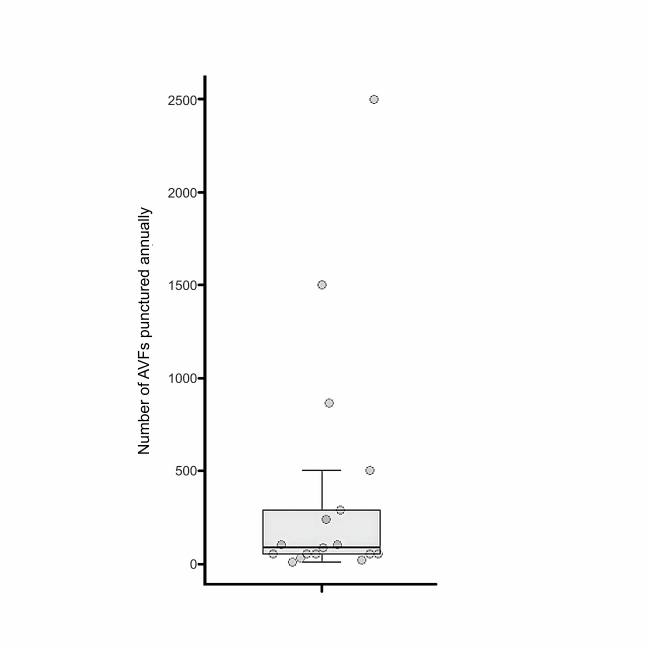
Number of arteriovenous fistula punctures per year, professionals from the Department of Renal Replacement Therapy, Fundação Hospital de Clínicas Gaspar Vianna, trained in arteriovenous fistula puncture. Belém, Pará, Brazil, 2024 Source: the author. AVF = arteriovenous fistula.

Figure 6 illustrates the variation in the number of punctures performed by each professional. Three professionals conducted from 863 to 2,500 AVF punctures per year, which are significantly higher volumes compared to the other study participants (the three points beyond the upper limit). However, the majority of participants are concentrated in the lower part of the graph, indicating that, in general, they conduct fewer punctures per year.

As shown in Table 3, 82% of the professionals did not perform AVF punctures with the aid of USG, and just 18% stated they used USG when conducting the procedure. Less than half of the professionals (29%) had had prior contact with simulators for AVF puncture training. Moreover, a majority stated they did not feel comfortable performing ultrasound-guided punctures (76%) and just a small proportion (12%) considered themselves skilled at using USG. Although the majority of these professionals work at private institutions (59%), the estimated mean proportion of USG-guided punctures in relation to the total number of punctures was low (0.5%), indicating that use of USG is still infrequent in practice.

**Table 3:** Initial characteristics of professionals from the Department of Renal Replacement Therapy, Fundação Hospital de Clínicas Gaspar Vianna, trained in arteriovenous fistula puncture. Belém, Pará, Brazil, 2024.

**Variables**	**n (%)**
Conducts punctures with USG	
Yes	3 (18%)
No	14 (82%)
Previous contact with the simulator (training)	
Yes	5 (29.4%)
No	12 (70.5%)
Skilled in USG	
Yes	2 (12%)
Partially	2 (12%)
No	13 (76%)
Comfortable performing ultrasound-guided puncture	
Yes	14 (82%)
No	1 (6%)
Did not answer	2 (12%)
Sector in which works most	
Private	10 (59%)
Public	6 (35%)
Both	1 (6%)
Estimated average proportion of punctures performed with USG guidance in relation to total number of AVF punctures performed to date (%)	0.5
Total	17 (100%)

USG = ultrasonography; AVF = arteriovenous fistula. Source: the author.

**Figure 7:**
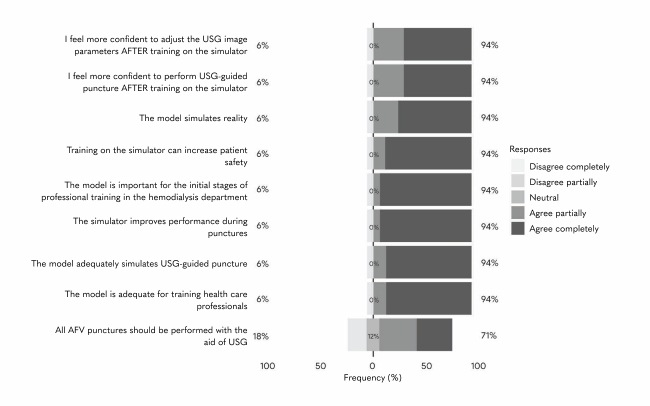
Assessment results, professionals from the Department of Renal Replacement Therapy, Fundação Hospital de Clínicas Gaspar Vianna, trained in arteriovenous fistula puncture with the ultrasound guided puncture simulator. Belém, Pará, Brazil, 2024 Source: the author. AVF = arteriovenous fistula; USG = ultrasonography.

Around 94% of the professionals agreed that training with simulators increased their confidence with adjusting the USG image parameters and performing ultrasound-guided puncture. They also considered that the simulation model emulates reality adequately and is important in the initial stages of training. Furthermore, they stated that simulator training improves performance during punctures and that the model is adequate for training health professionals. A significant proportion (71%) agreed that all AVF punctures should be performed with the aid of USG; although 12% were unsure and 18% disagreed with this statement (Figure 7).

**Figure 8:**
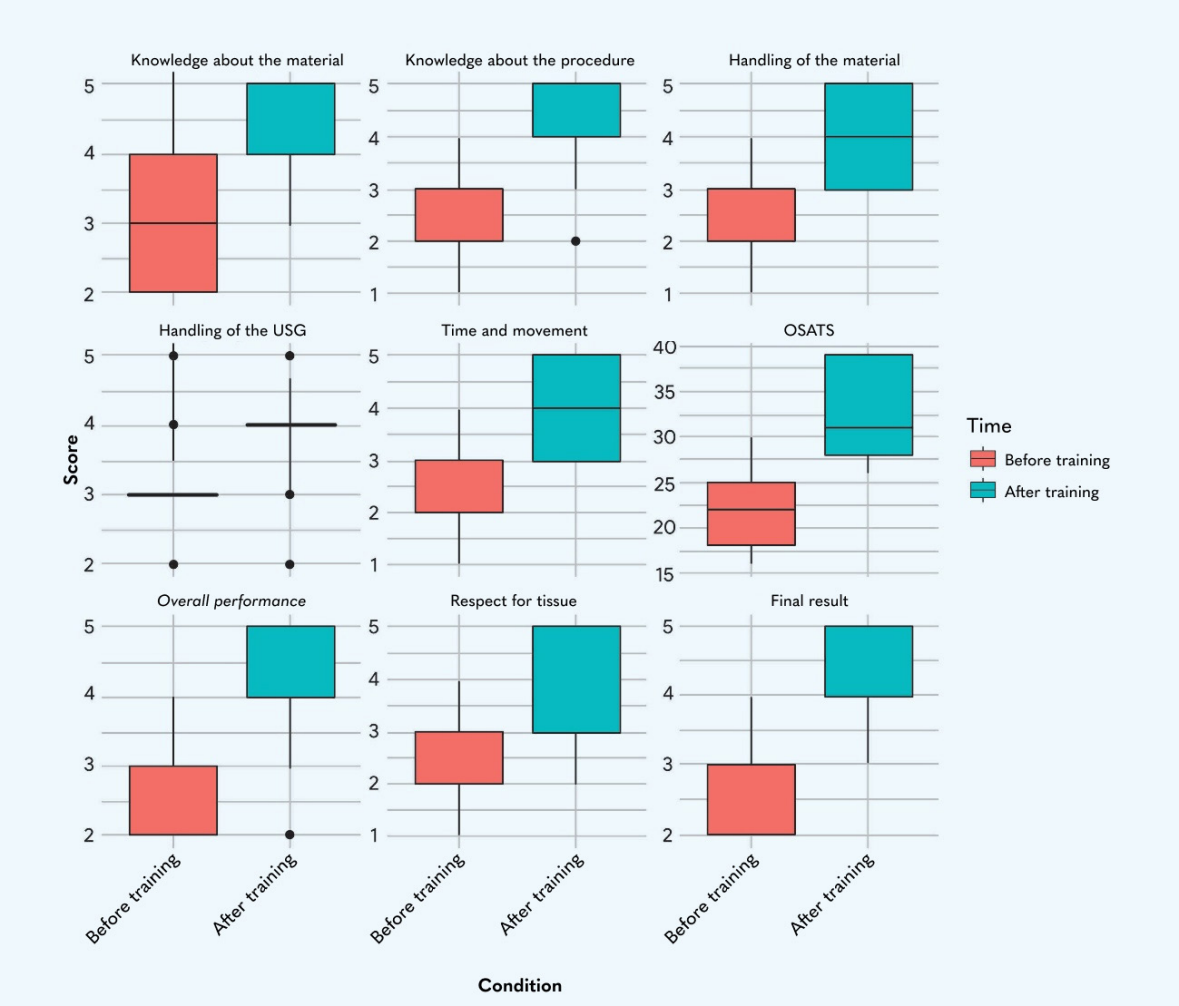
Comparison of performance before and after training with ultrasound guided puncture simulator, professionals from the Department of Renal Replacement Therapy, Fundação Hospital de Clínicas Gaspar Vianna, trained in arteriovenous fistula puncture. Belém, Pará, Brazil, 2024 Source: the author. USG = ultrasonography; OSATS = Objective Structured Assessment of Technical Skills.

Median scores for all of the items assessed (knowledge about the material, knowledge about the procedure, handling of the material, handling of the USG, time and movement, Objective Structured Assessment of Technical Skills [OSATS] score, overall performance, respect for tissue, and final result) were higher after training, indicating a general improvement in the professionals’ performance. The amplitude of the box plots, which reflects the distribution of the professionals’ data, tended to reduce after training, suggesting greater uniformity in the professionals’ performance after training. The most obvious improvement was observed in time taken to conduct the procedure (Figure 8).

The proportions before training suggests variability in the professionals’ experience or technical preparation, whereas the results after training reveal greater uniformity of results. This shows that the training can minimize discrepancies between the professionals, promoting greater safety and efficacy during procedures.

The improved OSATS scores highlight the impact of training on overall performance analyses, indicating that the simulator is an effective tool for improving technical skills and increases professionals’ confidence.

All of the items analyzed revealed significant improvement after training, as shown by the p values < 0.05. This shows that the training was effective for development of the professionals’ skills. It can be observed that the median (Md) increased for all items, revealing a general trend for higher scores after training, while the interquartile range (IQR) for four items (respect for tissue, time and movement, handling of the material, and OSATS score) revealed slightly larger differences at T2. This shows that the participants progressed at different speeds, since training confers new skills, and that the velocity or depth of learning may vary from one professional to the next (Table 4).

**Table 4:** Comparison of performance rated with the Objective Structured Assessment of Technical Skills score, at initial and final assessments (T1 and T2), professionals from the Department of Renal Replacement Therapy, Fundação Hospital de Clínicas Gaspar Vianna, trained in ultrasonography-guided arteriovenous fistula puncture.

**Specialist’s rating**	**Initial assessment (T1)**			**Final assessment(T2)**			
	**Md**	**Q25%-Q75%**	**IQR.**	**Md**	**Q25%-Q75%**	**IQR**	**P value**
**Respect for tissue**	2.0	(2.0-3.0)	1.0	3.0	(3.0-5.0)	2.0	0.006^*^
**Time and movement**	2.0	(2.0-3.0)	1.0	4.0	(3.0-5.0)	2.0	0.002*
**Knowledge about the material**	3.0	(2.0-4.0)	2.0	4.0	(4.0-5.0)	1.0	0.010*
**Handling of the material**	3.0	(2.0-3.0)	1.0	4.0	(3.0-5.0)	2.0	0.008*
**Handling of the USG**	3.0	(3.0-3.0)	0.0	4.0	(4.0-4.0)	0.0	0.026*
**Knowledge about the procedure**	3.0	(2.0-3.0)	1.0	4.0	(4.0-5.0)	1.0	< 0.001*
**Overall performance**	3.0	(2.0-3.0)	1.0	4.0	(4.0-5.0)	1.0	0.002*
**Final result**	3.0	(2.0-3.0)	1.0	4.0	(4.0-5.0)	1.0	0.001*
**OSATS**	22.0	(18.0-25.0)	7.0	31.0	(28.0-39.0)	11.0	< 0.001*

* Wilcoxon test.

Md = median; Q25%-Q75% = first and last quartiles; IQR = interquartile range; USG = ultrasonography; OSATS = Objective Structured Assessment of Technical Skills. Source: the author.

For all of the variables analyzed (respect for tissue, time and movement, knowledge about the material, etc.), the p values are much smaller than the conventional significance level of 0.05 (Table 4). This shows that there was a significant difference in the professionals’ performance after training with the simulator and that training had a positive effect on all of the variables assessed.

Ten months after the ultrasound-guided puncture course, the participants answered a questionnaire to assess knowledge retention. The results provide evidence about the lasting impacts of training with the simulator on these professionals’ practice (Table 5).

**Table 5:** Questionnaire on knowledge retention by professionals from the Department of Renal Replacement Therapy, Fundação Hospital de Clínicas Gaspar Vianna, 10 months after training in ultrasonography-guided arteriovenous fistula puncture with a simulator model.

**Variables**	**n (%)**
Simulator training should be performed regularly?	
Completely disagree	1 (5.8%)
Partially disagree	0
Neither disagree/nor agree	0
Agree partially	0
Completely agree	12 (70.5%)
Did not answer	4 (23.5%)
I feel more confident in performing AVF puncture with USG?	
Completely disagree	1 (5.8%)
Partially disagree	0
Neither disagree/nor agree	0
Agree partially	3 (17.6%)
Completely agree	9 (52.9%)
Did not answer	4 (23.5%)
I perform USG-guided AVF punctures as part of my daily practice?	
Yes	5 (29.4%)
No	8 (47%)
Did not answer	4 (23.5%)
Total	17 (100%)

AVF = arteriovenous fistula; USG = ultrasonography. Source: the author.

**Figure 9:**
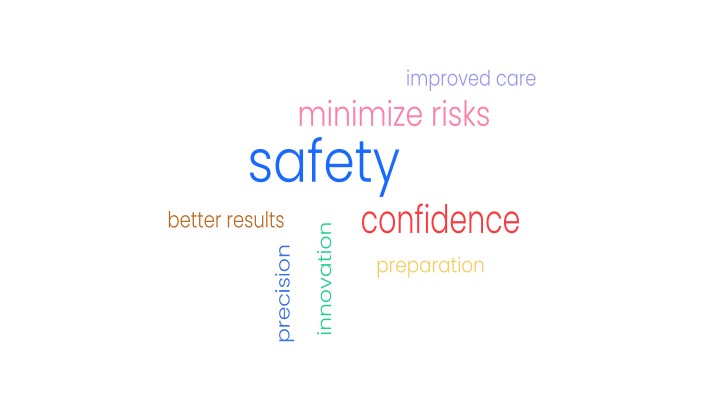
Most frequent responses of professionals from the Department of Renal Replacement Therapy, Fundação Hospital de Clínicas Gaspar Vianna, on the impact of training in ultrasonography-guided arteriovenous fistula puncture on their professional lives. Source: the author.

Moreover, when asked in a generic manner about the impact of the course on their daily professional routines, The most common responses are illustrated by the word cloud shown in Figure 9. The word used most often was “safety”, demonstrating the benefits of training with the simulator in terms of encouraging nurses to puncture with USG guidance, aligned with the care pillar of guaranteeing patient safety.

## DISCUSSION

This study analyzed the implementation of a professional training course for nurses at renal replacement therapy services, employing realistic simulation models and delivered at a teaching hospital in Pará (Brazil). The objective was to deliver an immersive theoretical and practical course based on simulation of ultrasound-guided puncture of AVFs and analyze the intervention’s impacts in terms of improvement of participants’ technical skills, offering a practical approach in a controlled environment.

Ultrasound-guided puncture of AVFs is a state-of-the-art technique that contributes to the durability and functionality of these structures, reducing complications such as hematoma, thrombosis, and infiltration. Studies suggest that there are positive results associated with use of USG for hemodialysis access in clinical practice.^12^ The discoveries made in research are promising, especially in relation to identification of possible access anomalies^15,16^ (such as pseudoaneurysms, presence of clots, tortuosity, and stenosis), facilitating routine and difficult cannulations,^15,17^ which could reduce punctures of the area and aneurysm formation, and reducing cannulation and needle handling errors, minimizing access damage. However, these findings must be interpreted with caution, given the limitations of the available data sources, comprising qualitative studies that did not specifically target the nursing team, which was the focus of the present study.

Simulation-based health care training is a field in rapid expansion, especially in the area of vascular access, in which it has gained widespread acceptance. This type of training is a response to growing social expectations and ethical commitments, since it leads to greater patient safety. Various types of simulators can yield significant improvements in development of technical knowledge, practical skills, communication, and even in the dynamics of team behavior. This active learning approach enables both basic skills and complex interventions to be practiced, offering professionals immersion in scenarios that reproduce real situations in clinical practice and contribute to safer and more effective training. They can be classified into two major groups: low and high fidelity simulations. The first are generally made using simpler materials and focus on basic skills, while the second are designed to reproduce a scenario or task in a more realistic manner and focus on acquisition of complex skills.

A confluence between recent events and potential benefits has resulted in greater applicability of these resources in health care education systems. These factors include the ability to shorten the learning curve for specific procedures to obtain proficiency in real cases. Simulation also enables provision of immediate feedback, adjusting the process steps of a procedure, and an objective and precise assessment of the professional’s performance, which is essential for professionals in training or specialists seeking professional advancement. Additionally, simulation-based training can have a global impact if taken to remote and resource-poor environments to train health professionals, while responding to calls for a new model of skills teaching focused on patient safety.

This study employed a sample comprising 17 nurses, the majority of whom were over the age of 45 years and female (Figure 5 and Table 1), the majority working at private institutions. Although many had a great deal of previous experience with AVF punctures, they had never performed punctures guided by USG or had only done so a few times (Table 3).

A review article published previously had already shown that a minority of professionals who work with chronic kidney patients reported using USG for punctures (just 13.6%).^18^ Some of the barriers to adoption mentioned were: the extra time needed to use USG for assessment and puncture, the limited availability of devices, professionals’ reluctance to use new technologies, and the possibility that USG could have a significant impact on workflow in already overloaded hemodialysis units.^19^

However, it has been reported in literature that when USG-guided cannulation is performed by trained professional, it takes just 2 minutes longer than blind puncture^20^ and actually has the potential to reduce complications such as incorrect cannulation, needle misalignment, and leakage; to detect needle infiltration in the vessel wall in the absence of signs or symptoms; to identify anomalies that are not visible at the skin surface; and to detect other puncturable areas of the vessel.^18^ As such, USG-guided cannulation reduces the frequency of complications and increases access longevity.

When prior ability with USG was surveyed, 76% of the professionals reported not having this skill and around 70% of them had never trained with a simulator. Many different simulation models have been documented in the literature as methods for training healthcare professionals in central venous access,^21-25^ peripheral access,^26^ and even for other types of surgery, such as laparoscopy,^27^ using the most diverse types of materials, from the most expensive to the most accessible.

For the present study, we chose a simple simulation model, albeit a very faithful and low-cost one, using a chicken breast containing a latex balloon filled with USG gel wrapped in plastic film, costing an average of 25 Reais per simulator. However, it is worth pointing out that the USG machines were not included in the research budget, since the HCGV DRRT already had machines available. Similar models, although with minor variations, have been described in the literature,^28,29^ and increases in venous cannulation success rates and reductions in the time taken for puncture were observed after training with these simulators.^29^

One important concern with regard to studies based on simulation is that they generally test proficiency on the simulator. Irrespective of this, there is good evidence showing that training with simulators improves technical skills and has the potential to improve performance in real interventions. Additionally, the course was organized because of the nursing professionals’ aspiration to discuss and improve their performance in USG-guided AVF procedures, especially with regard to better understanding of the techniques for USG-guided punctures.

As the body of literature on simulation-based medical education grows exponentially, the number of formats and assessment tools available to educators also grows. The OSATS score was developed and validated in 1996 at the University of Toronto as an instrument to effectively measure acquisition of technical skills for surgery. The OSATS score is made up of seven specific items reflecting different tasks, each one rated on a five-point scale. This structure allows calculation of an overall score for each item and an overall classification score. Over the years, the OSATS score has been adapted for different areas of health care, demonstrating its flexibility and utility for quantitative assessment of professionals’ technical competence.

In the present study, scores from 1 (very poor) to 5 (superior) were awarded for all of the OSATS items, before and after training, as follows: respect for tissue, time and movement, knowledge about the material, handling of the material, handling of the USG, knowledge about the procedure, and overall performance. After training with the simulator, participants exhibited better performance in all of these items, with statistically significant differences (p < 0.05) (Figure 8 and Table 4).

The improvement in OSATS scores and in the “final result” is particularly relevant, because it does not only reflect technical development, but also practical application and the quality of the results. This criterion, which is a standardized measure of technical performance, reveals one of the most significant improvements, highlighting the impact positive training had on general skills.

It is valid to note that this model afforded users the opportunity to practice until they felt comfortable and could be adapted to replicate several scenarios, such as different combinations of caliber and depth of veins, providing the professionals with practical learning.^28^ This observation was corroborated by the study participants, who rated the model as a good simulation of reality and as important for professional training and reported feeling more confident after training (Figure 7).

As part of this effort to achieve professional improvement, this study proposed to implement a training course in ultrasound-guided puncture of AVFs using a realistic simulator at the FHCGV, which is a center of excellence in renal replacement therapy in the Brazilian state of Pará. This realistic and immersive experience gave the participants the opportunity to hone their technical skills, to achieve a better understanding of a USG unit’s resources, and to build their confidence in undertaking these procedures. The fact that the study was a local experience enabled analysis not only of the technical and professional aspects, but also of its feasibility and applicability in a setting with limited resources, providing a model for other health centers in similar regions.

Notwithstanding, it is important to point out that this model also has certain important limitations. First is the fact that this model is perishable and the USG gel does not adequately simulate the texture of blood. Second is the absence of different scenarios in terms of the depth or tortuosity characteristic of AVFs. Finally, the model also lacks pulsating flow to simulate physiological conditions more realistically. Additionally, it is also valid to draw attention to the challenges inherent in implementation of simulation-based training, such as the costs and the need for basic teaching infrastructure and trained instructors. Despite these limitations, the model proved a useful aid for professional development and offered a more solid foundation in knowledge and practical skills, guaranteeing that these professionals are better prepared to face a variety of clinical conditions related to the difficulties inherent to cannulation of AVFs.

## CONCLUSIONS

Training ultrasound-guided puncture with a low-cost simulation model proved an effective means of training professionals from the renal replacement therapy department, improving their performance in all of the variables analyzed and making them more confident in performing USG-guided punctures. Its realistic characteristics confers the potential to foster long-term skills retention.

## Appendix A Termo de consentimento livre e esclarecido.

PUNÇÃO ECOGUIADA DE FÍSTULAS ARTERIOVENOSAS PARA HEMODIÁLISE: EXPERIÊNCIA DE UM CENTRO DE REFERÊNCIA NO PARÁ.

Você está sendo convidado a participar como voluntário do projeto de pesquisa acima citado. O documento abaixo contém todas as informações necessárias sobre a pesquisa que estamos fazendo. Sua colaboração nesta pesquisa será de muita importância para nós, mas se desistir a qualquer momento, isso não causará nenhum prejuízo a você. Fique ciente que não receberá remuneração e nenhum tipo de recompensa no decorrer da pesquisa, sendo sua participação voluntária. A proposta em estudo consiste na elaboração de um curso com treinamento em punção ecoguiada de fistulas arteriovenosas desenvolvido para profissionais que frequentemente lidam com punçãode FAVs. Você deve estar ciente: I) Participar desse projeto não lhe causará nenhum gasto com relação aos procedimentos efetuados com o estudo. II) Não será submetido a nenhum tratamento invasivo; São direitos seus: I) Responder ou não as perguntas contidas no instrumento de coleta dos dados da pesquisa; II) Desistir ou interromper a colaboração nesta pesquisa no momento em que desejar, sem necessidade de qualquer explicação, sem penalização e sem prejuízo a sua saúde ou bem estar físico; III) A indenização por danos decorrentes da participação na pesquisa deve estar de acordo com a Resolução CNS 466/12 - cobertura material para reparação a dano, causado pela pesquisa ao participante da pesquisa; IV) Garantia de Ressarcimento: Item 2.21 da Resolução CNS 466/12 – compensação material, exclusivamente de despesas do participante e seus acompanhantes, quando necessário, tais como transportes e alimentação; V) Benefícios: Os benefícios para os pesquisadores envolve o incremento de seus conhecimentos sobre o tema em questão e aplicação de estratégias para melhoria e redução dos riscos relacionados as complicações estudadas, servindo de modelo para os demais, como um exercício de integração entre pesquisa, ensino e saúde para um serviço de saúde pública mais eficiente. Aos pacientes, benefícios incluem a possibilidade de melhoria no procedimento de punção de FAVs durante hemodiálise, reduzindo intercorrências. VI) Riscos: Em relação aos riscos aos participantes da pesquisa, caberá o risco de sentirem-se desconfortáveis pela possibilidade de ter sua identidade e avaliações técnicas reveladas durante o tempo da pesquisa. As informações obtidas durante a pesquisa não terão o nome dos pesquisados. Além disso, valerá o comprometimento ético dos pesquisadores em manter a confidencialidade das informações, bem como a privacidade de seus conteúdos. VII) Decidir se sua identidade será divulgada e quais são, dentre as informações que forneceu as que podem ser tratadas de forma pública, com divulgação dos resultados da pesquisa em publicações científicas; VII) Ter garantida a confidencialidade das informações pessoais, assegurando sua privacidade; IX) Se desejar poderá pessoalmente, ou por telefone, entrar em contato com o pesquisador responsável para tomar conhecimento dos resultados parciais e finais desta pesquisa. Serviço de Cirurgia Vascular, FONE (091) 98242-0026. X) Caso desejar poderá também entrar em contato com o Comitê de Ética em Pesquisa-CEP da Fundação Pública Estadual Hospital das Clínicas Gaspar Vianna – FPEHCGV, que fica localizado no endereço: Trav. Alferes Costa n° 2000, Bairro: Pedreira, 1° andar, situado na Gerência de Ensino e Pesquisa, CEP: 666.087.-660, Belém-Pará, Contato: (91) 4005-2676, Email:comitedeetica@gasparvianna.pa.gov.br, Horário de atendimento: 08:00 às 14:00 (2ª a 6ª feira). Os CEPs foram criados para defender os direitos e interesses dos participantes das pesquisas, em sua integridade e dignidade, e para contribuir com o desenvolvimento das pesquisas dentro dos padrões éticos. XI) Receber uma via rubricada (em todas as páginas) e assinada do TCLE, pelo(s) pesquisador(res); Tendo recebido todos os esclarecimentos acima citados e ciente de meus direitos, concordo em participar desta pesquisa, bem como autorizo a divulgação e a publicação dos resultados em periódicos, revistas, apresentação em congressos, workshop e quaisquer eventos de caráter científico. Dessa forma, rubrico todas as páginas e assino este termo, juntamente com o pesquisador, em duas vias, de igual teor, ficando uma via sob meu poder e outra em poder do pesquisador.

( ) Desejo conhecer os resultados desta pesquisa.

( ) Não desejo conhecer os resultados desta pesquisa.

Belém, _______de _________________ de 2023. _________________________________________

Assinatura do Participante da Pesquisa

## Appendix B Características demográficas do grupo de especialistas envolvidos no curso de simulação.

**Table:**

**Características demográficas do grupo**	
**Nome do participante**	
**Sexo**	
**Idade**	
**Tempo de atuação com punção de FAVs**	
**Quantos casos (estimativa) de punção de fistulas arteriovenosas / ano?**	
**Quantos casos de punção já realizou guiado por USG? (estimativa na carreira)**	
**Contato prévio no simulador (treinamento)?**	
**Habilidades com USG previamente?**	
**Confortável com punção ecoguiada?**	
**Setor de maior atuação (público / privado)**	
**Estimativa (em %) de realização de punção guiada por USG em relação ao total de punções de FAV já realizadas.**	

## Appendix C Questionário para avaliação do simulador de punção de FAV ecoguiado.

**Table:**

**AFIRMATIVAS**	**Dircordo Plenamente**	**Dircordo Parcialmente**	**Nao discordo**	**Concordo Parcialmente**	**Concordo Totalmente**
			**Nao concordo**		
	**1**	**2**	**3**	**4**	**5**
1. O modelo simula a realidade.					
2. O modelo é adequado para treinamento de profissionais de saúde					
3. O modelo simula adequadamente a punção ecoguiada					
4. O simulador melhora o desempenho durante as punções					
5. O modelo é importante nas etapas inicias de formação do profissional no setor de hemodiálise					
6. O treino em simulador pode aumentar a segurança do paciente					
7. Me sinto mais seguro para realizar a punção ecoguiada após treino em simulador					
8. Me sinto mais seguro para ajustar os parâmetros de imagem no USG após treino em simulador					
9. TODAS as punções de FAV devem ser realizadas com auxilio de USG					

Comentários e sugestões: ____________________________________________________________________________________________________________________________________________________________________________________________________________________________________________________________________________________________________________________

Avaliador: ___________________________________ Data: ____________________

## Appendix D Escala de pontuação geral para simulação em punção ecoguiada.

**Table:**

**Código do aluno: Procedimento: Data:___/___/___**					
**Escala de Pontuação Geral**					

**Respeito pelo tecido**	1 Frequentemente usou força desnecessária e causou danos por uso inadequado do material	2	3 Manuseio cuidadoso dos tecidos, mas ocasionalmente ocorreu dano inadvertido	4	5 Abordagem adequada do material e dos tecidos, com dano mínimo
**Movimento e tempo**	1 Faz movimento desnecessário	2	3 Movimento eficazes, com algumas manobras desnecessárias	4	5 Clara economia de movimentos, com máxima eficiência
**Conhecimento do material**	1 Frequentemente pede material incorreto ou faz uso inapropriado do material	2	3 Sabe o nome da maior parte do material e usa-o de maneira adequada	4	5 Obviamente familiarizado com o material e seus nomes
**Manipulação do material**	1 Movimentos grosseiros e imprecisos, com perda do acesso, pouca estabilidade do material e inadequado posicionamento da agulha	2	3 Manobras competentes com quase nenhuma perda de acesso. Moderada estabilidade do material e bom posicionamento da agulha	4	5 Movimentos precisos, com estabilidade do material, manutenção dos acessos e perfeito posicionamento da agulha
**Manipulação do USG**	1 Não ajusta corretamente os parâmetros do USG para punção venosa	2	3 Ajusta parcialmente os parâmetros	4	5 Ajusta adequadamente os parâmetros
**Conhecimento do procedimento**	1 Conhecimento insuficiente. Inseguro e hesitante	2	3 Conhecia todos os passos importantes	4	5 Demonstra familiaridade com todos os passos
**Performance geral**	1 Muito ruim	2	3 Competente	4	5 Superior
**Resultado final**	1 Muito ruim	2	3 Competente	4	5 Superior

## Appendix E Questionário de retenção de conhecimento.

**Table:**

**AFIRMATIVAS**	**DISCORDO PLENAMENTE**	**DISCORDO PARCIALMENTE**	**NÃO DISCORDO**	**CONCORDO PARCIALMENTE**	**CONCORDO TOTALMENTE**
			**NÃO CONCORDO**		
	**1**	**2**	**3**	**4**	**5**
1. O treino em simuladores deve ser realizado periodicamente?					
2. Me sinto mais autoconfiante em realizar punção de fav guiada por USG?					

1. Já realizo punção ecoguiada de FAV na minha prática diária?

( ) sim ( )não

2. Qual foi o impacto do curso de punção de FAV guiado por USG na sua fica profissional?

________________________________________________________________________________________________________________________________________________________________________________________________
