# Supplementary material for: Training with ultrasound-guided puncture simulator of arteriovenous fistula for hemodialysis: experience from a center of excellence in Pará, Brazil
Source: J Vasc Bras. 2026 Mar 16;24:e20240184. doi: 10.1590/1677-5449.202401842 (PMC13007339; doi:10.1590/1677-5449.202401842)
Supplement: Translation

# Translation: Treinamento em simulador de punção ecoguiada de fístulas arteriovenosas para hemodiálise: experiência de um centro de referência no Pará, Brasil

**DOI:** 10.1590/1677-5449.202401841

**Authors:** Elisa Maria Novaes Barros, Glauco dos Santos Melo, Murilo Vasconcelos de Oliveira, Victor Hugo Guerreiro Americo Gomes, Gabriel Henrique de Paiva Ramos, Silvio Jorge de Oliveira Bentes, Humberto Balbi Reale, José Maciel Caldas dos Reis

**Affiliations:** 1 Fundação Hospital de Clínicas Gaspar Vianna – FHCGV, Belém, PA, Brasil.; 2 Hospital Beneficente Portuguesa – HBP, Belém, PA, Brasil.

**Keywords:** ultrassonografia, exercício de simulação, fistula arteriovenosa, diálise, enfermagem

## Abstract

**Contexto:**

A fístula arteriovenosa e o enxerto arteriovenoso são formas importantes de acesso para pacientes submetidos à hemodiálise. No entanto, a falha em canular com êxito um novo acesso arteriovenoso para um uso consistente ocorre em aproximadamente 20 a 40% das fístulas arteriovenosas. Dessa forma, a habilidade de realizar punção guiada por ultrassonografia se faz necessária, principalmente para acessos considerados difíceis. Contudo, a minoria dos profissionais de enfermagem se considera apta na manipulação da ultrassonografia. Assim, um ambiente de prática simulada permite que o treinando desenvolva suas habilidades e destreza para a manipulação simultânea da sonda e a inserção da agulha.

**Objetivos:**

Analisar os impactos do treinamento com simulador de acesso ecoguiado para fístulas arteriovenosas em um centro de referência em Belém, Pará.

**Métodos:**

Neste estudo analítico de intervenção, foram coletados dados referentes à equipe de enfermagem (17 enfermeiros) antes e após o treinamento de punção ecoguiada, utilizando um modelo de simulação com peito de frango e balão de látex, escolhido por seu baixo custo e elevada fidedignidade. O curso foi realizado em 26 e 27 de janeiro de 2024 na Fundação Hospital de Clínicas Gaspar Vianna.

**Resultados:**

Após o treinamento com simulador, os participantes tiveram um desempenho melhor em todas as variáveis analisadas, com diferença estatisticamente significativa. Ademais, avaliaram o modelo como um bom simulador da realidade, importante para o treinamento profissional, e relataram sentir-se mais confiantes após o treinamento.

**Conclusões:**

Os resultados indicam que o treinamento melhorou significativamente as habilidades dos profissionais de hemodiálise.

## INTRODUÇÃO

A fístula arteriovenosa (FAV) é um acesso arteriovenoso (AAV) fundamental para pacientes dialíticos, sendo a FAV autóloga considerada ideal para o acesso vascular a longo prazo^1,2^ .

A falha em canular um novo AAV, permitindo um uso consistente que idealmente deve ser livre de cateter, ocorre em aproximadamente 20 a 40% das FAVs. Isso se deve principalmente à trombose e à falha de maturação, enquanto a falha na primeira canulação, a isquemia e a infecção estão entre as causas menos frequentes^3-7^ .

A experiência do paciente durante a canulação do AAV é um fator crucial para o uso sustentado e eficaz do acesso. Assim, pacientes que necessitam de múltiplas tentativas de inserção de agulha ou que sofrem com infiltração grave, sangramento ou formação de hematoma apresentam níveis aumentados de insatisfação e dependência prolongada do uso de cateter venoso central, além de maior custo da assistência médica devido a testes e intervenções diagnósticas adicionais^8^ .

A ultrassonografia (USG), enquanto modalidade de imagem, apresenta diversas vantagens: é não invasiva, possui custo relativamente baixo e não requer contraste iodado nem o uso de radiação em sua rotina. No entanto, sua eficácia é limitada por ser uma técnica dependente do operador. Nesse cenário, a canulação guiada por USG, especialmente para o implante de cateteres, já é considerada nível IA de evidência^9^ . Contudo, no contexto da punção de FAV, é mais provável que seja empregada nos casos considerados difíceis, que podem incluir uma nova FAV em um paciente idoso, um acesso com histórico de múltiplas tentativas de canulação, um vaso de pequeno calibre, proximidade com estruturas como artéria ou nervo, ou um vaso cuja canulação na primeira tentativa seja crítica^10^ . Entretanto, na nefrologia, particularmente a praticada no Brasil, a integração da USG na avaliação clínica ainda é pouco difundida. As principais barreiras são o custo dos aparelhos e a falta de tempo para aprender a utilizar a USG^11^ .

Para um uso eficaz da USG, é necessário que o operador receba treinamento adequado, que inclua conhecimento teórico básico, além de instruções práticas de operadores experientes sobre o manuseio do equipamento e os procedimentos de canulação. O treinamento formal, como parte do currículo de enfermagem renal, deve resultar em um uso bem-sucedido da USG e na segurança do paciente^12^ . Assim, um ambiente de prática simulada permite que o treinando desenvolva suas habilidades e destreza para a manipulação simultânea da sonda e a inserção da agulha. Já foi demonstrado que o treinamento simulado melhora os resultados da educação em saúde e é valioso em outros aspectos da canulação vascular.

Adicionalmente, é importante destacar que a assistência ao paciente renal crônico na região amazônica enfrenta particularidades significativas, incluindo baixa escolaridade, renda insuficiente, doenças preexistentes e dificuldades no acesso a serviços de saúde, que representam vulnerabilidades críticas^13^ . A incorporação de tecnologias modernas, como a USG para a punção de FAVs e outros procedimentos em setores de hemodiálise, demonstra que, apesar dos desafios logísticos e de infraestrutura, serviços de excelência podem ser prestados na região.

Dessa forma, nesta pesquisa, analisamos os impactos do treinamento com um simulador de acesso ecoguiado para FAVs em um centro de referência em Belém, Pará.

## MÉTODO

### Desenho do estudo

Trata-se de um estudo observacional, transversal, descritivo e analítico. A metodologia aplicada seguiu os preceitos contidos no documento *Good Publication Practice Guidelines*, elaborado pelo Committee on Publication Ethics.

Durante a intervenção, foram coletados dados referentes à equipe de enfermagem (17 enfermeiros) antes e após a realização do curso de treinamento prático de punção ecoguiada em modelos de simulador de FAV para hemodiálise, ocorrido nos dias 26 e 27 de janeiro de 2024, nas dependências da Fundação Hospital de Clínicas Gaspar Vianna (FHCGV), localizado no município de Belém, Pará, no Setor de Terapia Renal Substitutiva (STRS) (Figura 1)**.**

**Figura 1:** Treinamento de punção ecoguiada dos profissionais de enfermagem do Setor de Terapia Renal Substitutiva, Fundação Hospital de Clínicas Gaspar Vianna. Fonte: acervo dos autores.

### Aspectos éticos

Este projeto foi aprovado pelo Comitê de Ética em Pesquisa da FHCGV, registrado na Plataforma Brasil sob o Certificado de Apresentação de Apreciação Ética: 48063521.0.0000.0016, e conforme parecer nº 5.412.470. Todos os participantes do estudo assinaram um Termo de Consentimento Livre e Esclarecido antes de ingressarem no estudo, conforme Apêndice A.

### População do estudo

Foram incluídos todos os profissionais do STRS da FHCGV que voluntariamente se interessaram em participar do treinamento de simulação de punção. Foram excluídos os profissionais que se recusaram a participar do estudo e aqueles que não atuavam com pacientes dialíticos, conforme fluxograma baseado nas diretrizes *Strengthening the Reporting of Observational Studies in Epidemiology*, demonstrado na Figura 2.

**Figura 2:** Fluxograma dos profissionais incluídos no estudo. Fonte: Elaborado pelos autores.

O modelo de treinamento foi um experimento realístico, não humano e de baixo custo, desenvolvido utilizando peito de frango com pele, balão de látex com gel de USG em seu interior e plástico filme envolvendo todo o conjunto (Figura 3). Na USG, obteve-se a imagem de uma estrutura tubular, de conteúdo anecóico e homogêneo dentro da musculatura do peito de frango, com densidade ultrassônica semelhante à do tecido muscular humano e com diâmetros compatíveis aos encontrados em FAVs na população dialítica (Figura 4), simulando com alta fidedignidade a imagem ultrassonográfica em humanos.

**Figura 3:** Modelo de punção com peito de frango e balão de látex tubular preenchido com gel de ultrassom. Fonte: acervo dos autores.

**Figura 4:** Imagem ultrassonográfica do modelo de punção. Fonte: acervo dos autores.

### Cálculo amostral

Para descrever os impactos do treinamento com um simulador realístico de acesso ecoguiado para FAVs em um centro de referência em Belém, foi realizado cálculo amostral para uma população, considerando um nível de confiança de 95% (Z = 1,96), erro tolerável de 5% e proporção esperada de 50%. Com base na equipe de enfermagem (enfermeiras do TRS), considerando um total de 22 profissionais no serviço, o tamanho amostral ideal seria de aproximadamente 21 participantes. No entanto, é importante contextualizar o delineamento do presente estudo: trata-se de uma pesquisa intervencionista, com aplicação de treinamento prático em um grupo específico e fechado de profissionais, cujo foco não é a generalização dos resultados para outras populações, mas sim a avaliação do efeito direto da intervenção dentro da realidade local do serviço estudado. Nessa perspectiva, a amostra de 17 participantes representa 77% da população total de interesse, o que confere ao estudo caráter quase censitário. O cálculo seguiu a metodologia proposta por Luchesa & Chaves Neto em 2011^14^ .

### Intervenção

Cada participante da pesquisa foi seu próprio controle, ou seja, foi comparado o desempenho do mesmo participante antes e após o treinamento. Os dados iniciais de *performance* no simulador de punção ecoguiada foram comparados à avaliação final, de acordo com a dinâmica do estudo, que foi distribuída em fases (primeira e segunda) e contemplou um componente teórico inicial, um curso prático de imersão e uma avaliação final e de retenção tardia.

A primeira fase consistiu em um curso teórico e prático com duração de 2 dias. Os treinandos tiveram a possibilidade de realizar as punções de acessos ecoguiados inicialmente de forma autônoma (momento inicial), posteriormente de forma monitorada por especialistas experientes, com *feedbacks* e ajustes das etapas processuais do procedimento, e, finalmente, realizaram novamente o procedimento de forma autônoma (momento final). Ao término do curso, foram preenchidos os protocolos de pesquisa com as variáveis a serem avaliadas (Apêndices B, C e D), que incluíram:

1. Dados demográficos;
2. Avaliação da experiência prévia dos profissionais do STRS com punção ecoguiada de FAVs;
3. Opinião dos profissionais quanto ao treinamento com simulador;
4. *Performance* do profissional pré e pós-treinamento com simulador.

Posteriormente, decorridos 10 meses da realização do curso, foi aplicado um novo questionário avaliando a retenção do conhecimento a longo prazo desses profissionais, assim como a análise subjetiva do impacto do curso na vida profissional dos participantes (Apêndice E).

### Análise estatística

Os dados foram tabulados em planilhas do Excel e descritas as características de nossa população de estudo, utilizando porcentagens para variáveis demográficas. O teste estatístico de Wilcoxon foi utilizado, sendo considerado estatisticamente significativo o valor de p < 0,05.

## RESULTADOS

Os participantes da pesquisa são majoritariamente do sexo feminino, conforme mostra a Figura 5. A partir da Tabela 1, verificamos que 65% dos profissionais estão na faixa etária de 33 a 45 anos ou mais, com média de idade de 28,8 anos.

**Figura 5:** Distribuição por sexo dos profissionais do Setor de Terapia Renal Substitutiva, Fundação Hospital de Clínicas Gaspar Vianna, habilitados em punção de fístulas arteriovenosas. Belém, Pará, 2024. Fonte: elaborado pela autora.

**Tabela 1:** Distribuição da idade dos profissionais do Setor de Terapia Renal Substitutiva, Fundação Hospital de Clínicas Gaspar Vianna, habilitados em punção de fístulas arteriovenosas. Belém, Pará, 2024.

**Faixa Etária**	**Quantidade**	**Porcentagem**
27 a 33	6	35%
33 a 39	1	6%
39 a 45	3	18%
45 ou mais	7	41%
Total	17	100%

Fonte: Elaborado pela autora.

O tempo médio de atuação dos profissionais é de 12,3 anos, o que significa que, em média, os profissionais têm mais de uma década de experiência nessa atividade. Entretanto, o desvio-padrão é de 8,4 anos, o que indica uma dispersão relativamente alta nos dados. Ou seja, existe grande variação no tempo de experiência entre os profissionais, com alguns tendo pouquíssima experiência e outros muitos anos de atuação. O tempo mínimo de atuação é de 6 meses, enquanto o máximo é de 25 anos, evidenciando uma ampla faixa de experiência entre os profissionais (Tabela 2).

**Tabela 2:** Medidas de tendência central e dispersão dos profissionais do Setor de Terapia Renal Substitutiva, Fundação Hospital de Clínicas Gaspar Vianna, habilitados em punção de fístulas arteriovenosas, segundo o tempo de atuação por ano. Belém, Pará, 2024.

**Variável**	**Média**	**Mediana**	**Desvio-padrão**	**Mínimo**	**Máximo**
Tempo de atuação com punção de FAVs (por ano)	12,3	14,0	8,4	0,6	25,0

FAVs = fístulas arteriovenosas. Fonte: Elaborado pela autora.

A Figura 6 ilustra a variabilidade na quantidade de punções realizadas por cada profissional. Três profissionais realizam entre 863 e 2.500 punções de FAV por ano, sendo este um número significativamente maior em comparação aos demais (pontos acima do limite superior). Porém, a maioria se concentra na parte inferior do gráfico, indicando que, em geral, realizam um número menor de punções por ano.

**Figura 6:** Quantidade de punções de fístulas arteriovenosas/ano, dos profissionais do Setor de Terapia Renal Substitutiva, Fundação Hospital de Clínicas Gaspar Vianna, habilitados em punção de fístulas arteriovenosas. Belém, Pará, 2024. Fonte: Elaborado pela autora. FAV = fístula arteriovenosa.

Segundo a Tabela 3, 82% dos profissionais não realizam a punção de FAVs com o auxílio de USG, e apenas 18% relatam utilizar a USG para realizar o procedimento. Menos da metade dos profissionais (29%) teve contato prévio com simuladores para treinamento da punção de FAVs. Além disso, a maioria relatou não se sentir confortável em realizar a punção ecoguiada (76%), e apenas uma pequena parcela (12%) se considera habilidosa com a USG. Apesar de a maioria dos profissionais atuar em instituições privadas (59%), a estimativa média de punções guiadas por USG em relação ao total de punções realizadas é baixa (0,5%), indicando que o uso da USG ainda é pouco frequente na prática.

**Tabela 3:** Características prévias dos profissionais do Setor de Terapia Renal Substitutiva, Fundação Hospital de Clínicas Gaspar Vianna, habilitados em punção de fístulas arteriovenosas. Belém, Pará, 2024.

**Variáveis**	**n (%)**
Punção realizada com USG	
Sim	3 (18%)
Não	14 (82%)
Contato prévio no simulador (treinamento)	
Sim	5 (29,4%)
Não	12 (70,5%)
Habilidades com USG	
Sim	2 (12%)
Parcialmente	2 (12%)
Não	13 (76%)
Confortável em realizar a punção ecoguiada	
Sim	14 (82%)
Não	1 (6%)
Não respondeu	2 (12%)
Setor de maior atuação	
Privado	10 (59%)
Público	6 (35%)
Ambos	1 (6%)
Estimativa média (em %) de realização de punção guiada por USG em relação ao total de punções de FAV já realizadas	0,5
Total	17 (100%)

USG = ultrassonografia; FAV = fístula arteriovenosa. Fonte: Elaborado pela autora.

Cerca de 94% dos profissionais concordam que o treinamento em simulador aumenta a segurança para ajustar os parâmetros de imagem na USG e para realizar a punção ecoguiada. Também consideram que o modelo do simulador reproduz a realidade de forma adequada e que é importante nas etapas iniciais de formação. Ainda relatam que o treinamento em simulador melhora o desempenho durante as punções e que o modelo é adequado para o treinamento de profissionais de saúde. Uma parcela significativa (71%) concorda que todas as punções de FAV devem ser realizadas com o auxílio de USG; entretanto, 12% não souberam responder e 18% discordaram da afirmação (Figura 7).

**Figura 7:** Respostas da avaliação dos profissionais do Setor de Terapia Renal Substitutiva, Fundação Hospital de Clínicas Gaspar Vianna, habilitados em punção de fístulas arteriovenosas, sobre o simulador de punção de fístula arteriovenosa ecoguiado. Belém, Pará, 2024. Fonte: Elaborado pela autora. FAV = fístula arteriovenosa; USG = ultrassonografia.

Todos os itens avaliados (conhecimento do material, conhecimento do procedimento, manipulação do material, manipulação de USG, movimento e tempo, escore *Objective Structured Assessment of Technical Skills* [OSATS], *performance* geral, respeito pelo tecido e resultado final) apresentaram mediana superior após o treinamento, indicando uma melhora geral no desempenho dos profissionais. A amplitude dos *boxplots*, que reflete a distribuição dos dados dos profissionais, tende a ser menor após o treinamento, sugerindo uma maior homogeneidade no desempenho dos profissionais após a capacitação. A melhora mais evidente foi observada no tempo de realização do procedimento (Figura 8).

**Figura 8:** Comparação de *performance* antes e após o treinamento com simulador de punção de fístulas arteriovenosas ecoguiado dos profissionais do Setor de Terapia Renal Substitutiva, Fundação Hospital de Clínicas Gaspar Vianna, habilitados em punção de fístulas arteriovenosas. Belém, Pará, 2024. Fonte: Elaborado pela autora. USG = ultrassonografia; OSATS = *Objective Structured Assessment of Technical Skills*.

A proporção parcial antes do treinamento sugere uma variabilidade na experiência ou na preparação técnica dos profissionais, enquanto os resultados após o treinamento mostram maior uniformidade nas respostas. Isso demonstra que o treinamento pode minimizar discrepâncias entre os profissionais, promovendo maior segurança e eficácia nos procedimentos.

A melhoria no escore OSATS destaca o impacto do treinamento em análises globais de desempenho, indicando que o simulador é uma ferramenta eficiente para melhorar habilidades técnicas e aumentar a confiança dos profissionais.

Todos os itens analisados mostraram uma melhoria significativa após o treinamento, como demonstrado pelo p < 0,05. Isso indica que o treinamento foi eficaz no desenvolvimento das habilidades dos profissionais. Observou-se um aumento na mediana (Md) de todos os itens, o que indica uma tendência geral para notas mais altas após a realização do treinamento, enquanto a distância interquartil (D. Interq.) em quatro itens (respeito pelo tecido, movimento e tempo, manipulação do material e OSATS) apresenta diferenças um pouco maiores no T2. Isso significa que os participantes progrediram em ritmos diferentes, pois o treinamento proporciona novas habilidades, e a velocidade ou profundidade do aprendizado pode variar entre os profissionais (Tabela 4).

**Tabela 4:** Comparativo do desempenho conforme o *Objective Structured Assessment of Technical Skills*, nos momentos inicial e final (T1 e T2), dos profissionais do Setor de Terapia Renal Substitutiva, Fundação Hospital de Clínicas Gaspar Vianna, em punção de fístulas arteriovenosas guiadas por ultrassonografia.

**Avaliação especialista**	**Momento inicial (T1)**			**Momento final (T2)**			
	**Md**	**Q25%-Q75%**	**D.Interq.**	**Md**	**Q25%-Q75%**	**D.Interq.**	**Valor de p**
Respeito pelo tecido	2,0	(2,0-3,0)	1,0	3,0	(3,0-5,0)	2,0	0,006^*^
Movimento e tempo	2,0	(2,0-3,0)	1,0	4,0	(3,0-5,0)	2,0	0,002*
Conhecimento do material	3,0	(2,0-4,0)	2,0	4,0	(4,0-5,0)	1,0	0,010*
Manipulação do material	3,0	(2,0-3,0)	1,0	4,0	(3,0-5,0)	2,0	0,008*
Manipulação do USG	3,0	(3,0-3,0)	0,0	4,0	(4,0-4,0)	0,0	0,026*
Conhecimento do procedimento	3,0	(2,0-3,0)	1,0	4,0	(4,0-5,0)	1,0	< 0,001*
*Performance* geral	3,0	(2,0-3,0)	1,0	4,0	(4,0-5,0)	1,0	0,002*
Resultado final	3,0	(2,0-3,0)	1,0	4,0	(4,0-5,0)	1,0	0,001*
OSATS	22,0	(18,0-25,0)	7,0	31,0	(28,0-39,0)	11,0	< 0,001*

* Teste de Wilcoxon.

Md = mediana; Q25%-Q75% = primeiro e último quartil; D.Interq = diferença interquartil; USG = ultrassonografia; OSATS = *Objective Structured Assessment of Technical Skills*. Fonte: Elaborado pela autora.

Para cada uma das variáveis analisadas (respeito pelo tecido, movimento e tempo, conhecimento do material etc.), o valor de p é muito menor do que o nível de significância convencional de 0,05 (Tabela 4). Isso significa que houve uma diferença significativa no desempenho dos profissionais após o treinamento em simulador e que o treinamento teve um efeito positivo sobre todas as variáveis avaliadas.

Após 10 meses da realização do curso de punção ecoguiada, os participantes responderam ao questionário de retenção de conhecimento. Neste, é possível evidenciar os impactos tardios do treinamento com simulador na prática desses profissionais (Tabela 5).

**Tabela 5:** Questionário de retenção de conhecimento dos profissionais do Setor de Terapia Renal Substitutiva, Fundação Hospital de Clínicas Gaspar Vianna, após 10 meses do treinamento de punção de fístulas arteriovenosas guiadas por ultrassonografia em modelo de simulador.

**Variáveis**	**n (%)**
Treino em simulador deve ser realizado periodicamente?	
Discordo plenamente	1 (5,8%)
Discordo parcialmente	0
Não discordo/não concordo	0
Concordo parcialmente	0
Concordo plenamente	12 (70,5%)
Não respondeu	4 (23,5%)
Me sinto mais autoconfiante em realizar punção de FAV com USG?	
Discordo plenamente	1 (5,8%)
Discordo parcialmente	0
Não discordo/não concordo	0
Concordo parcialmente	3 (17,6%)
Concordo plenamente	9 (52,9%)
Não respondeu	4 (23,5%)
Realizo punção de FAV ecoguiada na minha prática diária?	
Sim	5 (29,4%)
Não	8 (47%)
Não respondeu	4 (23,5%)
Total	17 (100%)

FAV = fístula arteriovenosa; USG = ultrassonografia. Fonte: Elaborado pela autora.

Apenas cerca de 30% dos profissionais que participaram do curso atualmente realizam punções ecoguiadas na prática diária. Contudo, ao serem questionados sobre sua autoconfiança para realizar tais punções, cerca de 70% concordaram parcial ou plenamente, sugerindo que a baixa prática diária não está relacionada à falta de capacidade técnica do profissional, mas sim a fatores como a indisponibilidade de aparelhos de USG ou a superlotação dos serviços de hemodiálise.

Além disso, quando perguntados de forma genérica sobre o impacto do curso no dia a dia do profissional, as respostas mais frequentes foram exemplificadas na nuvem de palavras da Figura 9. A palavra “segurança” foi a mais referenciada, demonstrando os benefícios do treinamento com simulador no encorajamento dos enfermeiros na punção guiada por USG e alinhando-se ao pilar de garantir a segurança ao paciente.

**Figura 9:** Respostas mais frequentes dos profissionais do Setor de Terapia Renal Substitutiva, Fundação Hospital de Clínicas Gaspar Vianna, sobre os impactos do treinamento de punção de fístulas arteriovenosas guiadas por ultrassonografia em suas vidas profissionais. Fonte: Elaborado pela autora.

## DISCUSSÃO

Este estudo avaliou a implementação de um curso de aprimoramento profissional para enfermeiros dedicados a serviços de terapia renal substitutiva, utilizando simuladores realísticos, em um hospital-escola no Pará. O objetivo foi implementar um curso de imersão teórico e prático baseado em simulação para punção ecoguiada de FAVs e analisar os impactos dessa intervenção na melhoria das habilidades técnicas dos participantes, oferecendo uma abordagem prática em um ambiente controlado.

A punção ecoguiada de FAVs é uma técnica de ponta que contribui para a longevidade e a funcionalidade dessas estruturas, reduzindo complicações como hematomas, trombose e infiltração. Estudos sugerem que resultados positivos estão associados ao uso de USG para acesso de hemodiálise na prática clínica^12^ . As descobertas das pesquisas incluídas são promissoras, especialmente em relação à identificação de possíveis anomalias de acesso^15,16^ (como pseudoaneurismas, presença de coágulo, tortuosidade e estenoses), facilitando canulações rotineiras e difíceis^15,17^ , que podem diminuir a punção da área e a formação de aneurisma, e reduzindo erros de canulação e manipulação de agulhas, minimizando danos ao acesso. No entanto, esses achados precisam ser interpretados com cautela, dadas as limitações da base de evidências existente, composta por estudos qualitativos e não direcionados especificamente para a equipe de enfermagem, avaliados no presente estudo.

O treinamento baseado em simulação na área da saúde representa um campo em franca evolução, especialmente na área de acessos vasculares, onde tem ganhado ampla aceitação. Esse tipo de treinamento atende às crescentes expectativas sociais e ao compromisso ético, pois implica garantir maior segurança aos pacientes. Simuladores variados proporcionam melhorias significativas no desenvolvimento de conhecimentos técnicos, habilidades práticas, comunicação e até mesmo nas dinâmicas de comportamento de equipe. Essa abordagem de aprendizado ativo permite a prática tanto de habilidades fundamentais quanto de intervenções complexas, promovendo uma imersão do profissional em cenários que reproduzem situações reais da prática clínica e contribuindo para uma formação mais segura e eficaz. Além disso, pode ser distribuída em dois grandes grupos: simulação de baixa e de alta fidelidade. A primeira é frequentemente composta por materiais mais simples e voltada a habilidades básicas, enquanto a segunda reproduz o cenário ou a tarefa de forma mais realística e é direcionada ao ganho de habilidades complexas.

Uma confluência de eventos recentes e potenciais benefícios levou a uma maior aplicabilidade desse recurso nos sistemas de educação em saúde. Esses fatores incluem a possibilidade de encurtar a curva de aprendizagem em procedimentos específicos para a obtenção de proficiência em casos reais. Além disso, a simulação permite o fornecimento de *feedback* imediato, com ajustes das etapas processuais do procedimento, e uma avaliação objetiva e precisa do desempenho do profissional, fato primordial para profissionais em formação ou especialistas em busca de aprimoramento profissional. Adicionalmente, o treinamento baseado em simulação pode ter um impacto global se levado a ambientes remotos e de poucos recursos para treinar profissionais de saúde e vai ao encontro do apelo por um novo modelo de aquisição de habilidades com foco na segurança do paciente.

A presente pesquisa contou com uma casuística de 17 enfermeiros, a maioria com mais de 45 anos e do sexo feminino (Figura 5 e Tabela 1), atuando majoritariamente em serviços privados. Apesar da grande experiência prévia com punção de FAVs, nunca haviam realizado punções guiadas por USG ou o haviam feito apenas raras vezes (Tabela 3).

Em artigo de revisão previamente publicado, tal cenário já havia sido demonstrado, em que a minoria dos profissionais que trabalham com renais crônicos relatava o uso de USG para punção (apenas 13,6%)^18^ . Dessa forma, algumas barreiras levantadas para tal cenário foram: o tempo extra que o uso da USG requereria para avaliação e punção, a disponibilidade limitada de dispositivos, a relutância do profissional em usar novas tecnologias e a possibilidade de que a USG tenha um impacto significativo no fluxo de trabalho já sobrecarregado nas unidades de hemodiálise^19^ .

Contudo, já é relatado na literatura que a canulação guiada por USG, quando realizada por profissional treinado, gasta apenas cerca de 2 minutos a mais do que a punção às cegas^20^ e tem, na verdade, potencial para: reduzir complicações como canulação incorreta, desalinhamento e extravasamento da agulha; detectar infiltração da agulha na parede do vaso na ausência de sinais ou sintomas; identificar anomalias não visíveis na superfície da pele; e detectar outras áreas do vaso puncionáveis^18^ . Dessa forma, a canulação guiada por USG reduz a frequência de complicações e aumenta a longevidade do acesso.

Quando avaliadas as habilidades prévias com o USG, 76% dos profissionais relataram não possuir tal habilidade, e cerca de 70% desses nunca sequer haviam treinado com simulador. Diversos modelos de simulação já foram documentados na literatura como meio de treinamento do profissional de saúde em acesso venoso central^21-25^ , periférico^26^ e até mesmo para outras cirurgias, como as laparoscópicas^27^ , utilizando os mais diversos materiais, desde os mais caros até os mais acessíveis.

No presente estudo, optamos por um modelo de simulação simples, porém bastante fidedigno e de baixo custo, que utiliza o peito de frango com um balão de látex preenchido por gel de USG e envolto por plástico filme, custando em média 25 reais por simulador. Contudo, é válido ressaltar que os aparelhos de USG não foram incluídos no orçamento da pesquisa, visto que o STRS do HCGV já possuía os aparelhos à disposição. Modelos similares, porém com pequenas variações, já foram descritos na literatura^28,29^ , e, após o treinamento com tais simuladores, foi notado um aumento das taxas de sucesso na canulação venosa e redução no tempo para punção^29^ .

Uma preocupação importante nos estudos baseados em simulação é que eles geralmente medem a proficiência no simulador. Independentemente disso, há boas evidências de que o treinamento em simulador melhora as habilidades técnicas e tem o potencial de aumentar o desempenho durante intervenções reais. Além disso, o curso foi organizado com base em uma aspiração dos profissionais de enfermagem para discutir e melhorar a *performance* nos procedimentos guiados por USG em FAVs, especialmente no melhor entendimento e nas técnicas para punções ecoguiadas.

À medida que a literatura sobre educação médica baseada em simulação cresce exponencialmente, também cresce o número de formatos e ferramentas de avaliação disponíveis para educadores. O OSATS foi desenvolvido e validado pela Universidade de Toronto em 1996 como um instrumento para mensurar de maneira eficiente a aquisição de habilidades técnicas em cirurgia. O OSATS é composto por sete itens específicos que refletem diferentes tarefas, cada um avaliado em uma escala de cinco pontos. Essa estrutura permite a geração de uma pontuação geral em cada item e de uma pontuação global de classificação. Ao longo dos anos, o OSATS tem sido adaptada para diferentes áreas da saúde, demonstrando sua flexibilidade e utilidade na avaliação quantitativa da competência técnica de profissionais.

No presente estudo, foram atribuídas pontuações de 1 (muito ruim) a 5 (superior) em todos os itens avaliados no OSATS, antes e após o treinamento, sendo estes: respeito pelo tecido, movimento e tempo, conhecimento sobre o material, manipulação do material e da USG, conhecimento do procedimento e *performance* geral. Em todos esses quesitos, após o treinamento com simulador, os participantes apresentaram melhor desempenho, com diferença estatisticamente significativa (p < 0,05) (Figura 8 e Tabela 4).

A melhora no escore OSATS e no “resultado final” é particularmente relevante, pois reflete não apenas o desenvolvimento técnico, mas também a aplicação prática e a qualidade dos resultados. Esse critério, que é uma medida padronizada da *performance* técnica, mostra uma das melhorias mais significativas, destacando o impacto positivo do treinamento nas habilidades gerais.

É válido ressaltar que este modelo fornece aos usuários a oportunidade de praticar até se sentirem confiantes e pode ser adaptado para replicar vários cenários, como diferentes tipos de calibre e profundidades de veias, proporcionando um aprendizado prático para os profissionais^28^ . Esse achado é corroborado pelos participantes da pesquisa, que avaliaram o modelo como um bom simulador da realidade, importante para o treinamento profissional, e relataram se sentir mais confiantes após o treinamento (Figura 7).

Como parte desse esforço de aprimoramento profissional, este estudo propôs a implementação de um curso de treinamento em punções ecoguiadas de FAVs em um simulador realístico na FHCGV, centro de excelência em terapia renal substitutiva no estado do Pará. A experiência realística e imersiva proporcionou aos participantes a oportunidade de aprimorar habilidades técnicas, garantir um melhor entendimento dos recursos da USG e aumentar a autoconfiança na realização do procedimento. O fato de o estudo ser uma experiência local permite avaliar não só os aspectos técnicos dos profissionais, mas também sua viabilidade e aplicabilidade em uma realidade de recursos limitados, servindo como modelo para outros centros de saúde em regiões semelhantes.

Contudo, é importante ressaltar que este modelo também apresenta algumas limitações importantes. Primeiro: o fato de o modelo ser perecível e o gel de USG não simular adequadamente a textura do sangue. Segundo: a ausência de diferentes cenários de profundidade ou tortuosidades característicos de FAVs. E, finalmente, a ausência de um fluxo pulsátil que simule condições fisiológicas mais realísticas. Além disso, é válido destacar os desafios inerentes à implementação do treinamento baseado em simulação, como o custo, a necessidade de infraestrutura básica de ensino e a capacitação dos instrutores. Apesar das limitações, o modelo se mostrou útil para auxiliar no aprimoramento profissional e proporcionar uma base mais sólida de conhecimentos e habilidades práticas, garantindo que os profissionais estejam melhor preparados para enfrentar uma variedade de condições clínicas relacionadas às dificuldades de canulação de FAVs.

## CONCLUSÃO

O treinamento de punção ecoguiada com modelo de simulação de baixo custo demonstrou ser eficiente para o treinamento de profissionais do setor de terapia renal substitutiva, melhorando o desempenho destes em todas as variáveis analisadas e tornando-os mais confiantes para punções guiadas por USG. Pelo caráter realístico, tem potencial de favorecer a retenção de habilidades em longo prazo.

## Apêndice A Termo de consentimento livre e esclarecido.

PUNÇÃO ECOGUIADA DE FÍSTULAS ARTERIOVENOSAS PARA HEMODIÁLISE: EXPERIÊNCIA DE UM CENTRO DE REFERÊNCIA NO PARÁ.

Você está sendo convidado a participar como voluntário do projeto de pesquisa acima citado. O documento abaixo contém todas as informações necessárias sobre a pesquisa que estamos fazendo. Sua colaboração nesta pesquisa será de muita importância para nós, mas se desistir a qualquer momento, isso não causará nenhum prejuízo a você. Fique ciente que não receberá remuneração e nenhum tipo de recompensa no decorrer da pesquisa, sendo sua participação voluntária. A proposta em estudo consiste na elaboração de um curso com treinamento em punção ecoguiada de fistulas arteriovenosas desenvolvido para profissionais que frequentemente lidam com punçãode FAVs. Você deve estar ciente: I) Participar desse projeto não lhe causará nenhum gasto com relação aos procedimentos efetuados com o estudo. II) Não será submetido a nenhum tratamento invasivo; São direitos seus: I) Responder ou não as perguntas contidas no instrumento de coleta dos dados da pesquisa; II) Desistir ou interromper a colaboração nesta pesquisa no momento em que desejar, sem necessidade de qualquer explicação, sem penalização e sem prejuízo a sua saúde ou bem estar físico; III) A indenização por danos decorrentes da participação na pesquisa deve estar de acordo com a Resolução CNS 466/12 - cobertura material para reparação a dano, causado pela pesquisa ao participante da pesquisa; IV) Garantia de Ressarcimento: Item 2.21 da Resolução CNS 466/12 – compensação material, exclusivamente de despesas do participante e seus acompanhantes, quando necessário, tais como transportes e alimentação; V) Benefícios: Os benefícios para os pesquisadores envolve o incremento de seus conhecimentos sobre o tema em questão e aplicação de estratégias para melhoria e redução dos riscos relacionados as complicações estudadas, servindo de modelo para os demais, como um exercício de integração entre pesquisa, ensino e saúde para um serviço de saúde pública mais eficiente. Aos pacientes, benefícios incluem a possibilidade de melhoria no procedimento de punção de FAVs durante hemodiálise, reduzindo intercorrências. VI) Riscos: Em relação aos riscos aos participantes da pesquisa, caberá o risco de sentirem-se desconfortáveis pela possibilidade de ter sua identidade e avaliações técnicas reveladas durante o tempo da pesquisa. As informações obtidas durante a pesquisa não terão o nome dos pesquisados. Além disso, valerá o comprometimento ético dos pesquisadores em manter a confidencialidade das informações, bem como a privacidade de seus conteúdos. VII) Decidir se sua identidade será divulgada e quais são, dentre as informações que forneceu as que podem ser tratadas de forma pública, com divulgação dos resultados da pesquisa em publicações científicas; VII) Ter garantida a confidencialidade das informações pessoais, assegurando sua privacidade; IX) Se desejar poderá pessoalmente, ou por telefone, entrar em contato com o pesquisador responsável para tomar conhecimento dos resultados parciais e finais desta pesquisa. Serviço de Cirurgia Vascular, FONE (091) 98242-0026. X) Caso desejar poderá também entrar em contato com o Comitê de Ética em Pesquisa-CEP da Fundação Pública Estadual Hospital das Clínicas Gaspar Vianna – FPEHCGV, que fica localizado no endereço: Trav. Alferes Costa n° 2000, Bairro: Pedreira, 1° andar, situado na Gerência de Ensino e Pesquisa, CEP: 666.087.-660, Belém-Pará, Contato: (91) 4005-2676, Email:comitedeetica@gasparvianna.pa.gov.br, Horário de atendimento: 08:00 às 14:00 (2ª a 6ª feira). Os CEPs foram criados para defender os direitos e interesses dos participantes das pesquisas, em sua integridade e dignidade, e para contribuir com o desenvolvimento das pesquisas dentro dos padrões éticos. XI) Receber uma via rubricada (em todas as páginas) e assinada do TCLE, pelo(s) pesquisador(res); Tendo recebido todos os esclarecimentos acima citados e ciente de meus direitos, concordo em participar desta pesquisa, bem como autorizo a divulgação e a publicação dos resultados em periódicos, revistas, apresentação em congressos, workshop e quaisquer eventos de caráter científico. Dessa forma, rubrico todas as páginas e assino este termo, juntamente com o pesquisador, em duas vias, de igual teor, ficando uma via sob meu poder e outra em poder do pesquisador.

( ) Desejo conhecer os resultados desta pesquisa.

( ) Não desejo conhecer os resultados desta pesquisa.

Belém, _______de _________________ de 2023. _________________________________________

Assinatura do Participante da Pesquisa

## Apêndice B Características demográficas do grupo de especialistas envolvidos no curso de simulação.

**Table:**

Características demográficas do grupo	
Nome do participante	
Sexo	
Idade	
Tempo de atuação com punção de FAVs	
Quantos casos (estimativa) de punção de fistulas arteriovenosas / ano?	
Quantos casos de punção já realizou guiado por USG? (estimativa na carreira)	
Contato prévio no simulador (treinamento)?	
Habilidades com USG previamente?	
Confortável com punção ecoguiada?	
Setor de maior atuação (público / privado)	
Estimativa (em %) de realização de punção guiada por USG em relação ao total de punções de FAV já realizadas.	

## Apêndice C Questionário para avaliação do simulador de punção de FAV ecoguiado.

**Table:**

**AFIRMATIVAS**	**Dircordo Plenamente**	**Dircordo Parcialmente**	**Nao discordo**	**Concordo Parcialmente**	**Concordo Totalmente**
			**Nao concordo**		
	**1**	**2**	**3**	**4**	**5**
1. O modelo simula a realidade.					
2. O modelo é adequado para treinamento de profissionais de saúde					
3. O modelo simula adequadamente a punção ecoguiada					
4. O simulador melhora o desempenho durante as punções					
5. O modelo é importante nas etapas inicias de formação do profissional no setor de hemodiálise					
6. O treino em simulador pode aumentar a segurança do paciente					
7. Me sinto mais seguro para realizar a punção ecoguiada após treino em simulador					
8. Me sinto mais seguro para ajustar os parâmetros de imagem no USG após treino em simulador					
9. TODAS as punções de FAV devem ser realizadas com auxilio de USG					

Comentários e sugestões: ____________________________________________________________________________________________________________________________________________________________________________________________________________________________________________________________________________________________________________________

Avaliador: ___________________________________ Data: ____________________

## Apêndice D Escala de pontuação geral para simulação em punção ecoguiada.

**Table:**

Código do aluno: Procedimento: Data:___/___/___					
Escala de Pontuação Geral					

Respeito pelo tecido	1 Frequentemente usou força desnecessária e causou danos por uso inadequado do material	2	3 Manuseio cuidadoso dos tecidos, mas ocasionalmente ocorreu dano inadvertido	4	5 Abordagem adequada do material e dos tecidos, com dano mínimo
Movimento e tempo	1 Faz movimento desnecessário	2	3 Movimento eficazes, com algumas manobras desnecessárias	4	5 Clara economia de movimentos, com máxima eficiência
Conhecimento do material	1 Frequentemente pede material incorreto ou faz uso inapropriado do material	2	3 Sabe o nome da maior parte do material e usa-o de maneira adequada	4	5 Obviamente familiarizado com o material e seus nomes
Manipulação do material	1 Movimentos grosseiros e imprecisos, com perda do acesso, pouca estabilidade do material e inadequado posicionamento da agulha	2	3 Manobras competentes com quase nenhuma perda de acesso. Moderada estabilidade do material e bom posicionamento da agulha	4	5 Movimentos precisos, com estabilidade do material, manutenção dos acessos e perfeito posicionamento da agulha
Manipulação do USG	1 Não ajusta corretamente os parâmetros do USG para punção venosa	2	3 Ajusta parcialmente os parâmetros	4	5 Ajusta adequadamente os parâmetros
Conhecimento do procedimento	1 Conhecimento insuficiente. Inseguro e hesitante	2	3 Conhecia todos os passos importantes	4	5 Demonstra familiaridade com todos os passos
Performance geral	1 Muito ruim	2	3 Competente	4	5 Superior
Resultado final	1 Muito ruim	2	3 Competente	4	5 Superior

## Apêndice E Questionário de retenção de conhecimento.

**Table:**

**AFIRMATIVAS**	**DISCORDO PLENAMENTE**	**DISCORDO PARCIALMENTE**	**NÃO DISCORDO**	**CONCORDO PARCIALMENTE**	**CONCORDO TOTALMENTE**
			**NÃO CONCORDO**		
	**1**	**2**	**3**	**4**	**5**
1. O treino em simuladores deve ser realizado periodicamente?					
2. Me sinto mais autoconfiante em realizar punção de fav guiada por USG?					

1. Já realizo punção ecoguiada de FAV na minha prática diária?

( ) sim ( )não

2. Qual foi o impacto do curso de punção de FAV guiado por USG na sua fica profissional?

________________________________________________________________________________________________________________________________________________________________________________________________
